# The contribution of theta and delta to feedback processing in children with developmental language disorder

**DOI:** 10.1186/s11689-023-09481-1

**Published:** 2023-04-17

**Authors:** Asiya Gul, Lauren S. Baron, Yael Arbel

**Affiliations:** MGH Institute of Health Professions, Boston, MA USA

## Abstract

**Purpose:**

The study aimed at evaluating feedback processing at the electrophysiological level and its relation to learning in children with developmental language disorder (DLD) to further advance our understanding of the underlying neural mechanisms of feedback-based learning in children with this disorder.

**Method:**

A feedback-based probabilistic learning task required children to classify novel cartoon animals into two categories that differ on five binary features, the probabilistic combination of which determined classification. The learning outcomes’ variance in relation to time- and time–frequency measures of feedback processing were examined and compared between 20 children with developmental language disorder and 25 age-matched children with typical language development.

**Results:**

Children with developmental language disorder (DLD) performed poorer on the task when compared with their age-matched peers with typical language development (TD). The electrophysiological data in the time domain indicated no differences in the processing of positive and negative feedback among children with DLD. However, the time–frequency analysis revealed a strong theta activity in response to negative feedback in this group, suggesting an initial distinction between positive and negative feedback that was not captured by the ERP data. In the TD group, *delta* activity played a major role in shaping the FRN and P3a and was found to predict test performance. *Delta* did not contribute to the FRN and P3a in the DLD group. Additionally, theta and *delta* activities were not associated with the learning outcomes of children with DLD.

**Conclusion:**

*Theta* activity, which is associated with the initial processing of feedback at the level of the anterior cingulate cortex, was detected in children with developmental language disorder (DLD) but was not associated with their learning outcomes. *Delta* activity, which is assumed to be generated by the striatum and to be linked to elaborate processing of outcomes and adjustment of future actions, contributed to processing and learning outcomes of children with typical language development but not of children with DLD. The results provide evidence for atypical striatum-based feedback processing in children with DLD.

## Introduction

Feedback processing is an essential part of learning, particularly for school-aged children who are expected to adjust behaviors and strategies following external feedback provided to them by educators. Feedback processing is particularly important for children with various learning disorders, such as those experienced by children with developmental language disorder (DLD), because intervention techniques rely heavily on the provision of feedback, and it is unclear to what extent children with this disorder benefit from feedback. Feedback processing also plays a role in language development. While language acquisition, particularly syntax, relies primarily on the ability to implicitly extract statistical regularities from the environment (e.g., [31, 72, 80, 81], evidence suggests that corrective feedback contributes to and facilitates such learning [26, 73]. For example, Saxton [73] demonstrated that, contrary to common assumptions that language learning does not involve corrective feedback by caregivers, parents naturally provide corrective feedback on syntactic errors committed by their children during development. Dale and Christiansen [26] further demonstrated that when corrective feedback is provided to typically developing children during an artificial grammar learning task, learning outcomes improve in comparison to artificial grammar learning without feedback. Taken together, although it is common to think that language acquisition has little to do with the ability to process feedback, corrective feedback facilitates learning, even in the context of language skills whose acquisition relies primarily on implicit processes.

DLD is a neurodevelopmental language disorder that cannot be attributed to hearing loss, low nonverbal intelligence, or other known neurological deficits [11, 15]. It has been proposed that children with DLD have difficulty acquiring language, and in particular grammatical structures of language, because of a general deficit in the ability to implicitly extract regularities (e.g., [84]. The suggestion that children with DLD have impaired implicit learning is supported by behavioral data [49, 64, 82]. Additionally, brain structures linked to implicit learning show abnormalities in individuals with DLD (e.g., [55, 56]. Theories such as the Procedural deficit hypothesis propose that because implicit learning is impaired in children with DLD, intervention should capitalize on declarative mechanisms [85]. Intervention approaches that are declarative in nature rely heavily on the provision of feedback. Interestingly, feedback processing hinges on the intactness of the frontal cortex (e.g. [33, 46], and the basal ganglia (e.g. [21, 74, 75, 83], that are also implicated in implicit learning.

It has been proposed that ineffective feedback processing contributes to the impaired learning observed in children with DLD (e.g., [1, 2, 57]. More specifically, when engaged in feedback-based learning, children with DLD have been shown to exhibit poor learning outcomes and differences in the electrophysiological markers of feedback processing, i.e., feedback-related negativity (FRN) and P3a event-related potential (ERP) components, when compared with their peers [2, 3, 6]. For example, in Arbel et al. [2], a two-choice paired-associate declarative learning task was employed in which children were tasked with learning the correct associations between novel objects and names. In this task, feedback was deterministic and informative on a trial-by-trial basis, as it either confirmed a correct choice or indicated that the alternative answer was correct. The learning outcomes of children with DLD were inferior to those of their age-matched peers. Additionally, no differences between the processing of positive and negative feedback at the electrophysiological level (i.e., in the amplitudes of the FRN and P3a) were observed in children with DLD. In Gul et al. [43], children were presented with a probabilistic classification learning task in which the probabilistic information provided by the feedback had to be accumulated over many trials for learning to occur. In this paradigm, performance and processing differences between children with DLD and their peers were also observed.

It has been suggested that the FRN is the time-domain manifestation of low-frequency oscillatory signals indexing different neural mechanisms, the striatum which is responsible for reward processing, and the anterior cingulate cortex which responds to unfavorable outcomes. The present report aims at evaluating the neurophysiological mechanism underlying the impaired feedback processing in children with DLD. While much has been learned about feedback processing in children with DLD by evaluating the FRN and P3a ERP components, a more nuanced examination of the neuro mechanisms involved in the processing of feedback can be achieved through a time–frequency analysis of the EEG signal.

Converging evidence from temporal-spatial principal component analysis (PCA) and time–frequency techniques suggests that the FRN is a composite signal superimposed by a gain-related *delta* (~ 1–3 Hz) stemming from neural generators within the striatum [10, 18, 36] and loss-related *theta* (~ 4–7 Hz) activity eliciting from the anterior cingulate cortex (ACC) [18, 35, 47, 71]. It has been suggested that gains lead to an increase in *delta*, and losses induce *theta* activity [13, 35, 89]. These activities overlap partially in time but are dissociable in frequency and linked to different processes [8, 14, 19, 24].

While the exact function of these time–frequency indices of feedback processing is still under debate, recent work indicates that *theta* activity indexes a mechanism of cognitive control [19, 20, 25] or a top-down error-processing system that facilitates communication between neural systems to signal a need to adjust behavior following negative feedbacks or losses [20, 25, 86]. The *delta* activity, on the other hand, has been associated with reward processing [35, 76, 78]. Within the feedback-based learning paradigms, theta activity is suggested to distinguish the most salient features, such as feedback valence, while *delta* is sensitive to more elaborated processing of secondary attributes of the stimulus, such as behavioral outcomes or the magnitude of the outcome [12, 35, 44, 89, 90]. Furthermore, recent work has reported a decrease in post-feedback *theta* power and an increase in *delta* power with age [17]. These observations are consistent with the developmental pattern of the ACC, which is the underlying neuronal mechanism of theta activity [67, 68] and with reports of age-related changes in the FRN [3, 6, 91], with larger FRN to negative feedback in children relative to adolescents and adults [3, 5, 32]. The connection between feedback-related *delta* and the striatum is strengthened by findings that individuals with dysfunctional striatum exhibit an atypical *delta* response to reward processing (e.g., [35]. The notion that both *theta* and *delta* activity uniquely contribute to the FRN and emerge from distinct neuronal mechanisms while being subjected to developmental changes makes the interpretation of time-domain FRN challenging, particularly in children and adolescents.

Within the context of DLD, there is uncertainty about the neurocognitive basis of the disorder, with some evidence showing abnormalities in the striatum [7, 50, 58, 60, 77, 79, 87, 88] and some pointing to frontal cortex abnormalities [22, 23, 28, 37, 41, 50, 51]. The neurophysiological mechanisms supporting feedback processing can shed light on the deficit underlying DLD by elucidating through methods of electrophysiological signal decomposition at the time and time–frequency domains the involvement of the two neural generators in the impaired feedback processing in DLD. The results may support the hypothesis that reward processing which relies on the striatum, is the core feedback-based learning deficit in DLD or strengthen the view of DLD as dominated by impairment in the detection of errors communicated through negative feedback at the ACC level.

### Current study

The purpose of this study was to evaluate the neural correlates of feedback processing in children with DLD using time–frequency measures in conjunction with the time-domain measures. Based on previous findings, we expected that the FRN and P3a would exhibit composite signals of overlapping activity in the *theta* and *delta* frequency bands [12, 13, 35, 36]. To investigate the relationship between neural measures (i.e., time- and time–frequency domain) and behavioral indicators of feedback-based learning, we aimed to evaluate valence- and outcome-related modulations in the FRN as well P3a and related *delta* and *theta* oscillatory activities. We expected that *theta* activity would be sensitive to primary response to feedback (i.e., discriminating negative from positive feedback) and that *delta* activity would correlate with behavioral outcomes [12, 89]. Atypical behavior of the *delta* activity in children with DLD would provide support to the hypothesis that DLD is associated with impaired striatum, while abnormal theta activity would implicate the ACC.

## Methods

### Participants

Forty-five children between the ages of 8 and 13 years (*M* = 10.28, SD = 1.49; 20 females) from the Boston area were recruited for the study. All participants were right-handed individuals with normal or corrected vision and no history of head injury, neurological deficits (e.g., history of seizures), or other neurodevelopmental disorders (e.g., ADD/ADHD, autism spectrum disorder). Twenty of the participants had developmental language disorder (DLD) (*M* = 10.64 years, SD = 1.75; 9 females), and 25 had typical language development (TD) (*M* = 10.02 years, SD = 1.26, 11 Females). Participant demographic data, standardized assessment scores, and group comparisons are presented in Table 1. The DLD and TD groups did not significantly differ in age or proportion of males and females (i.e., *p* > 0.05). Parental consent and participant assent were obtained before data collection was initiated, and participants were paid for their participation. All children had nonverbal intelligence skills above the range of intellectual disability (SS > 70) on the Matrices subtest of the Kaufman Brief Intelligence Test, 2nd Edition (KBIT-2). Children with DLD had a reported history of a delay in language development and persistent difficulties with verbal and/or written language. Additionally, to be included in the DLD group, children had to obtain a Core Language Score that is at least 1.2 SD below the mean (SS < 82) on the Clinical Evaluation of Language Fundamentals, 5th Edition (CELF-5) or an Identification Core Score (ICS) below the cut point for their age group (i.e., less than 34 if 8–11 years old or less than 42 if 12–18 years old) on the Test of Integrated Language and Literacy Skills (TILLS). Both the ICS and the more interpretable standard score equivalent are reported in Table 1. As expected, based on group classification criteria and the common neuropsychological profile of children with DLD (e.g., [15, 38], the TD group scored significantly higher on the CELF-5, TILLS, and KBIT-2. This study was approved by the Mass General Brigham Institutional Review Board.

**Table 1:** Participant demographic data, standardized assessment scores, and group comparisons

		DLD	TD	One-way ANOVA results		
		*N* = 20	*N* = 25	*df*	*F*	*p*
Age (in months)		142.60 (19.85)	124.76 (16.56)	1, 43	0.001	.977
KBIT-2 Matrices Score		98.85 (13.96)	115.36 (10.34)	1, 43	20.78	< .001
CELF-5 Core Language Score		83.47 (8.03)	112.20 (13.41)	1, 43	68.29	< .001
TILLS Identification Core Score ^a^		26.00 (8.64)	–	–	–	–
TILLS Identification Core Standard Score		67.25 (12.89)	–	–	–	–
				Chi-squared test results		
Sex:	Female Male	9 11	11 14	*df*	*χ*^*2*^	*p*
				1, *N* = 45	0.004	.947

Values are presented as mean (standard deviation); Assessment data are standard scores. *KBIT-2* = Kaufman Brief Intelligence Test, 2nd Edition [52]; *CELF-5* = Clinical Evaluation of Language Fundamentals, 5th Edition [92]; *TILLS* = Test of Integrated Language and Literacy Skills [63]

^a^The TILLS was administered to 8 participants, 5 of whom did not meet criteria for DLD based on CELF-5 performance but whose parents reported a history of language delay or impairment

### Procedure and task

After completing the standardized assessments, participants were fitted with a 32-channel EEG hydrocel net by Electrical Geodesics Inc. (EGI) and then seated in a quiet room at a comfortable distance (60 cm) from a computer monitor that was adjusted in height to align the center of the screen with the participant’s eye level. Each participant completed a probabilistic classification learning (PCL) task, lasting about 15 min, while their EEG data were recorded. The task contained a training phase, which was immediately followed by a testing phase. Items (stimuli and feedback) were presented on a computer monitor, and responses were recorded on a Chronos response box. No reading or verbal responses were required during the task.

**Fig. 1:**
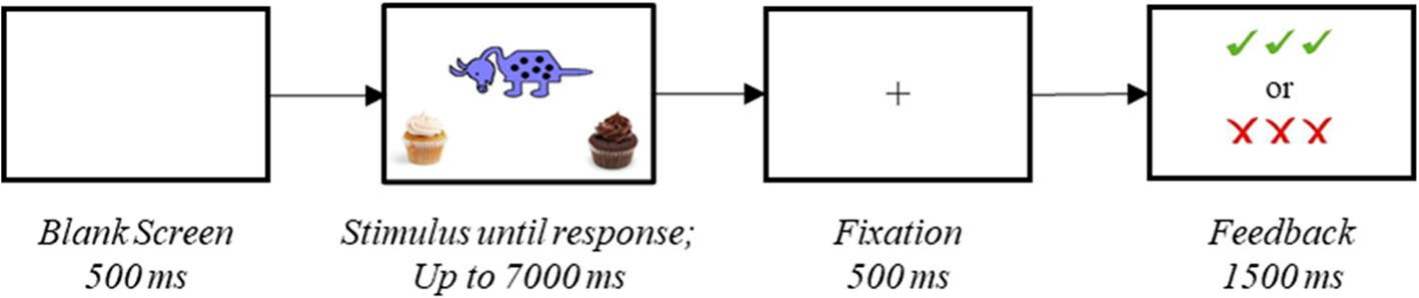
Trial structure for the probabilistic classification learning task

The experimental paradigm used in the present study was a simplified version of a probabilistic learning task to fit children [95]. In this task, children were required to classify novel cartoon animals into two categories that differ on five binary features (i.e., head position, tail shape, feet shape, body shape, body pattern), the probabilistic combination of which determined classification. Each of the five features had two possible versions, one of which was associated with each of the two classification categories. For instance, for the binary dimension “body shape”, the two options were “round” and “square,” with a round body being a *Category A* feature, and a squared body being a *Category B* feature. Feature distribution was based on a continuum, and the percentage contribution of these features determined classification. The task was split into two phases, a training phase and a test phase. During the training phase, participants were presented with eight novel animal exemplars repeated in a random order five times in each of four blocks for a total of 20 presentations of each exemplar during training and 160 total training trials. Each trial started with a blank screen for 500 ms, followed by a visual display of an animal exemplar in the center of the screen with the two category options at the bottom of the screen. Participants were instructed to press the right or left button based on the choice they make to classify the exemplar. Each stimulus was displayed until a response was registered (or up to 7 s), and then a fixation cross was shown for 500 ms. Feedback was provided in the form of three “√”s for correct responses and three “X”s for incorrect responses with a duration of 1500 ms. A visual summary of the trial structure is presented in Fig. 1. A detailed description of the task is provided in Gul et al. [43].

### EEG data acquisition

The Electrical Geodesics Inc. (EGI; Eugene, OR) System 400 with 32-channel HydroCel Geodesic Sensor Nets from EGI was used to obtain EEG data, comprised of Ag/AgCl electrodes attached to an elastic net following the international 10–20 system. EEG was continuously recorded at a 1000-Hz sampling rate. All electrode impedances were kept below 50 kΩ prior to data collection and used vertex as the reference electrode [34]. The presentation of stimuli was controlled by programmable experiment generation software (E-prime 2.0,Psychological Software Tools, Pittsburgh, PA), and signals were acquired across all electrodes. The offline EEG data processing and analysis were performed using custom MATLAB (The MathWorks, Inc., Natick, MA) scripts operating in conjunction with the open-source EEGLAB toolbox [27], http://sccn.ucsd.edu/eeglab). An Adaptive Mixture ICA (AMICA) was applied separately to a single subject dataset [66] to detect and correct eye movement and blinks. The data were then sorted into two feedback categories: negative feedback (NF) and positive feedback (PF). An evaluation of the proportion of trials rejected due to artifacts indicated that, on average, 5.42% of the PF trials (5% in the TD group; 5.93% in the DLD group) and 6.19% of the NF trials (4.48% in the TD group; 8.32% in the DLD group) were rejected. No *group *(TD, DLD*)* or *feedback type* (PF, NF) differences in the proportion of trials rejected were found (*p*s > 0.05). However, an interaction between *group* and *feedback type* was found, *F* (1, 43) = 4.619, *p* = 0.037, *ŋ*^*2*^ = 0.097. This interaction indicated that NF trials in the DLD group had the largest proportion of artifacts detected and rejected. It is possible that DLD responded with more body movement to negative feedback than to positive feedback, whereas children in the TD group did not show a similar pattern. The average number of artifact-free trials in the TD group was 91.80 (SD = 17.37) PF trials and 58.32 (SD = 13.67) NF trials. In the DLD group, the average number of artifact-free trials was 80.75 (SD = 17.40) PF trials and 65 (SD = 13.94) NF trials (see Table 2). All participants had a minimum of 30 trials per feedback type (positive and negative feedback), which is well above the minimum number of trials required to obtain a reliable error-related ERP component [65] and Cronbach’s alpha values above the threshold for high internal consistency according to Boudewyn et al. [16].

**Table 2:** Number of trials per group and feedback type

		**Number of trials per condition per group**		
		Number of trials	Number of trials	Number of rejected
		Before rejection	After rejection	Trials
**DLD**	NF	73.00 (12.50)	65.00 (13.94)	8.00 (10.14)
	PF	87.00 (12.50)	80.75 (17.40)	6.72 (9.61)
**TD**	NF	63.04 (15.46)	58.32 (13.67)	4.72 (5.10)
	PF	96.96 (15.46)	91.80 (17.37)	5.64 (6.32)

Values are presented as mean (standard deviation)

Note that ERP/ERSP analysis was performed on trials from the training session only, and both correct and incorrect trials were part of the analysis as the purpose of the study was to compare the processing of positive and negative feedback (i.e., performance feedback following correct and incorrect responses).

### EEG data analysis

Continuous EEG signals were down sampled to 250 Hz and filtered using a high-pass finite impulse response (FIR) at 0.5 Hz and a low-pass 40 Hz filter. Feedback-locked epochs were extracted as segments of 3 s (1 s before to 2 s after feedback presentation) and averaged separately for the negative and positive feedback. Each trial was visually inspected for movement artifacts that were manually removed following an automatic artifact removal with a ± 75 μV criterion. Visual inspection of the averaged data across electrodes indicated a maximal feedback-related activation at FCz. This observation is in line with previous reports of maximum FRN peak at FCz in adults (e.g., [35, 42, 54, 62] and children (e.g., [3, 32]. EEG data from electrode FCz were analyzed for time- and time–frequency domains separately.

#### Time-domain data analysis

ERP data from the fronto-central electrode of interest (FCz) were extracted for both the FRN and P3a, using EEGLab’s statistical tools for each participant and condition. The processed data were segmented into 1200 ms epochs extending 200 ms before and 1000 ms after feedback presentation. Each trial was visually inspected for movement artifacts which were manually removed. Baseline correction was performed on the averaged data, based on the signal in the 200 ms preceding the feedback stimulus (i.e., − 200 to 0 ms). Data were re-referenced to the average of all electrodes [94].

Temporal PCA (TPCA) was performed on the ERP datasets to reduce the temporal dimensionality of the dataset and to disentangle ERP components that overlapped in time (e.g., [5, 6] using Promax rotation [29]. The analysis used the covariance between time points and resulted in a set of eleven temporal factors accounting for 86.23% of the total variance. Temporal factor 6 (TF6) with a maximal peak around 250 ms and TF2 peaking around 400 ms were found to capture the FRN and P3a activation, respectively. The factor scores of TF6 and TF2 served as the amplitude measures of the FRN and P3a and were subjected to statistical analysis using IBM SPSS Statistics 24.0 (IBM, Armonk, NY). Significant effects were corrected for non-sphericity using Greenhouse–Geisser corrections, and significant effects are reported with the corrected degrees of freedom when appropriate.

#### Time–frequency analyses

EEG oscillatory activity induced by feedback processing was evaluated by computing event-related spectral perturbations (ERSP) and inter-trial coherence (ITC). The spectral power reflected in the event-related spectral perturbation (ERSP) and phase coherence shown by the inter-trial coherence (ITC) in conjunction with time-domain measures was extracted using custom MATLAB codes in conjunction with EEGLAB [27]. ERSP is an index of the relative change in EEG power at different frequencies with respect to stimulus onset. However, it may not give a temporally stable and precise measure across trials. The ITC is insensitive to EEG power but captures the temporal and spectral synchrony of the EEG signal across trials and reflects the extent to which the phase of neuronal activity elicited by task events is altered [27]. It reaches its maximum value of 1 for perfectly phase-aligned neural activity and becomes 0 when phase distribution becomes completely randomized. The ITC could be seen as a complementary measure to ERSP.

Baseline correction was performed on the averaged data, based on the signal within the 500 ms preceding the feedback stimulus (i.e., − 500 to – 300 ms). Data were re-referenced to the average [94]. To account for the trade-off between temporal and frequency resolution, newtimef() was used that applies Morlet wavelet transform to epoch time-series data such that with increasing frequencies, the wavelet keeps modifying [27]. Therefore, using newtimf(), average power was computed for the 90 linear-spaced frequencies ranging from 1 to 30 Hz along 300 linearly spaced time bins (1 cycle at the lowest frequency to 15 at the highest). Following the work of Foti et al. [35] and Bernat et al. [12], we extracted ERSP and ITC measures for the *delta* (1 to 3 Hz) and *theta* frequency range (4 to 7 Hz) during the FRN (200–400 ms) and P3a (300–500 ms) time ranges. ERSP and ITC values were averaged for each subject across trials.

### Statistical analysis

#### Behavioral data

To evaluate accuracy during training and test, a multi-variate ANOVA was conducted with *group* (TD, DLD) as a between-subjects variable.

#### ERP data

To examine differences in brain activation associated with feedback valence (i.e., positive and negative feedback) within and between groups (i.e., TD and DLD), a two-way repeated-measures ANOVA was conducted on the amplitudes of the FRN and P3a yielded by the TPCA from electrode FCz.

#### EEG time–frequency data

To evaluate the time–frequency measures of the FRN and the P3a between groups, a two (*feedback* valence: negative vs. positive) by two (*groups*: TD vs. DLD) repeated measures ANOVA was carried out for both ERSP and ITC measures. In the next step of the analysis, we evaluated the extent to which *delta* and mid-frontal *theta* activity contributed to the FRN and P3a components as suggested by previous work [12, 13, 35] and whether this contribution and its relation to learning outcomes differed across groups (TD, DLD). To evaluate the extent to which theta and *delta* activity uniquely contributed to the FRN and the P3a and whether there were any group differences, regression analyses were performed. Given that the *delta* and *theta* measures were each related to the time domain FRN and P3a measures separately, we assessed the exclusive effects of these time–frequency measures by entering them as simultaneous predictors in multiple linear regression. Four multiple linear regression models were used to examine variance in the time-domain TPCA factor measures of the FRN and P3a elicited by negative and positive feedback responses separately. Two predictors (*delta* and *theta* measures) to each feedback (negative and positive) were entered into each model simultaneously, with the time-domain FRN and P3a as the dependent variables. Specifically, FRN negative, FRN positive, P3a negative, and P3a positive ERP components served as the dependent variables; their corresponding *delta* and *theta* measures were used as independent variables.

**Table 3:** Results of regression models using ERSP measures of *delta* and *theta* to predict the FRN and P3a for group and feedback valence

**ERSP regression models**													
		**TD**						**DLD**					
		**Negative**			**Positive**			**Negative**			**Positive**		
**Model**		**Beta**	**t**	**Semi P. Corr**	**Beta**	**t**	**Semi P. Corr**	**Beta**	**t**	**Semi P. Corr**	**Beta**	**t**	**Semi P. Corr**
**FRN**	**Delta**	0.405	2.04	0.399	0.879	5.503^***^	0.761	0.381	1.496	0.341	0.224	0.87	0.206
	**Theta**	-0.407	-2.051	-0.401	-0.281	-1.758	-0.351	-0.359	-1.411	-0.317	0.298	1.159	0.271
	**F**	3.159			16.018^***^			1.439			2.293		
	**Adj. R2**	0.223			0.593			0.145			0.212		
**P3a**	**Delta**	0.523	3.187^**^	0.562	0.578	2.705^*^	0.5	0.285	1.217	0.283	0.1	0.474	0.114
	**Theta**	0.303	1.845	0.366	-0.116	0.545	-0.115	0.471	2.014	0.439	0.633	3.009^**^	0.59
	**F**	9.096^***^			4.207^*^			7.885^**^			7.887^**^		
	**Adj. R2**	0.453			0.277			0.481			0.481		

^*^Correlation is significant at the 0.05 level (2-tailed)

^**^Correlation is significant at the 0.01 level (2-tailed)

^***^Correlation is significant at the 0.001 level (2-tailed)

## Results

### Behavioral results

A significant main effect of *group* for training accuracy was revealed, *F* (1, 41) = 8.474, *p* = 0.006, *η*_*p*_^*2*^ = 0.171, indicating that accuracy during training was significantly higher for children in the TD group (*M* = 0.67, SD = 0.14) than for children in the DLD group (*M* = 0.56, SD = 0.14). Likewise, children with TD performed better than children with DLD on the test, *F* (1, 41) = 26.687, *p* < 0.001, *η*_*p*_^*2*^ = 0.394.

### Time-domain EEG results

**Fig. 2:**
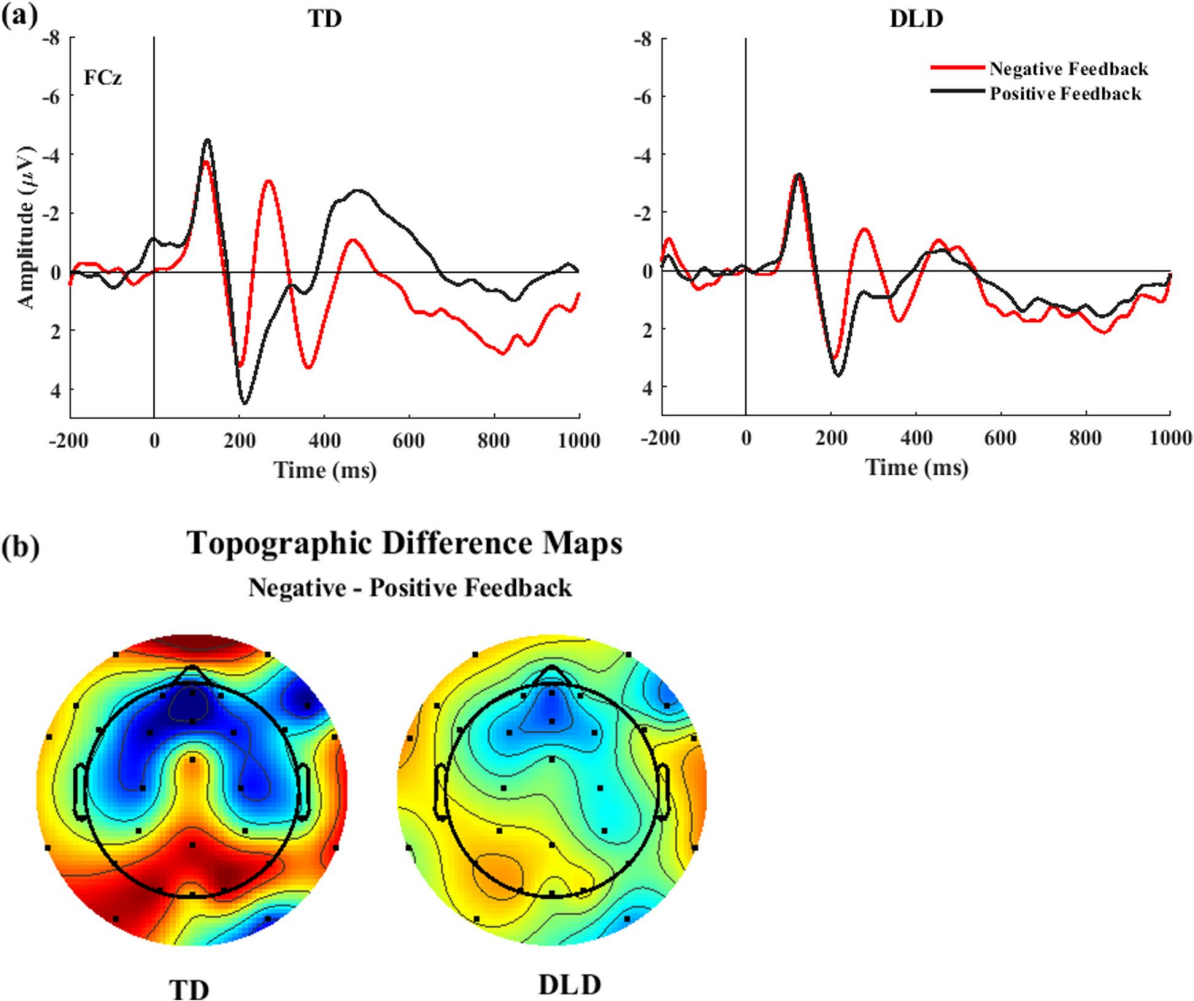
**a**. Grand-averaged ERPs time-locked to the onset of the feedback at electrode FCz for children with TD (on the left) and DLD (on the right). **b**. Topo maps in the FRN time window (220–280 ms) of the difference voltage between negative and positive feedback for children with TD (on the left) and children with DLD (on the right)

Figure 2 presents the ERPs captured in electrode FCz for each feedback type (positive, negative) and group (TD, DLD). A repeated measure ANOVA on the amplitude of the FRN yielded by TPCA revealed a main effect of *feedback valence*, *F* (1, 43) = 20.090, *p* < 0.001, *η2* = 0.318, indicating that the amplitude of the FRN to negative feedback was larger (more negative) than the amplitude of the FRN to positive feedback. No *group* effect was found, *F* (1, 43) = 0.981, *p* = 0.328, *η2* = 0.022; however, an interaction between *group* and *feedback valence* was detected, *F* (1, 43) = 4.671, *p* = 0.036, *η2* = 0.098. A follow-up *t* test revealed that FRN amplitude differences between negative and positive feedback were only present in the TD group (TD: *t(24)* =  − 5.095*, p* < 0.001; DLD: *t*(19) =  − 1.516, *p* = 0.145). Similar analysis on P3a revealed a main effect of *feedback valence*, *F* (1, 43) = 14.295, *p* < 0.001, *η2* = 0.249*,* indicating that the P3a elicited by negative feedback was larger than that elicited by positive feedback. Although there was no *group* effect, *F* (1, 43) = 0.369, *p* = 0.547, *η2* = 0.008, an interaction between *group* and *feedback* valence was found, *F* (1, 43) = 4.941, *p* = 0.032, *η2* = 0.103. Follow-up analysis using *t* tests indicated P3a amplitude differences between positive and negative feedback were only found in the TD group TD *t*(24) = 3.975, *p* < 0.001; DLD, *t*(19) = 1.304, *p* = 0.208.

### Time–frequency EEG results

**Fig. 3:**
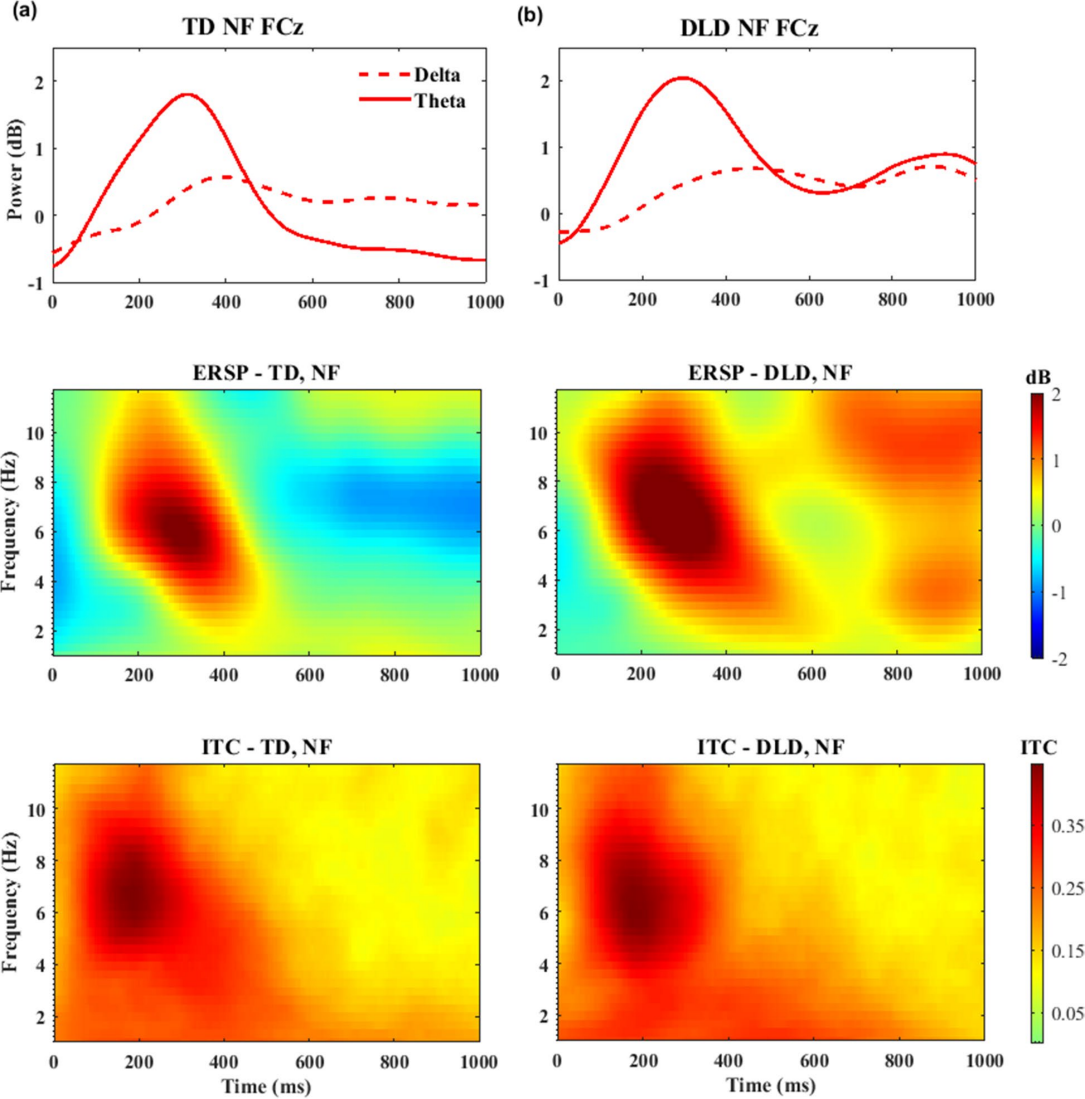
Time–frequency measures elicited by negative feedback (NF) at electrode FCz for children with TD (**a**) and DLD (**b**). On the top are filtered time–frequency waveforms; dotted lines represent the power of the delta frequency band (<3 Hz), and solid lines represent the theta frequency band (4–7 Hz). ERSP and ITC measures are presented in the middle and at the bottom, respectively

**Fig. 4:**
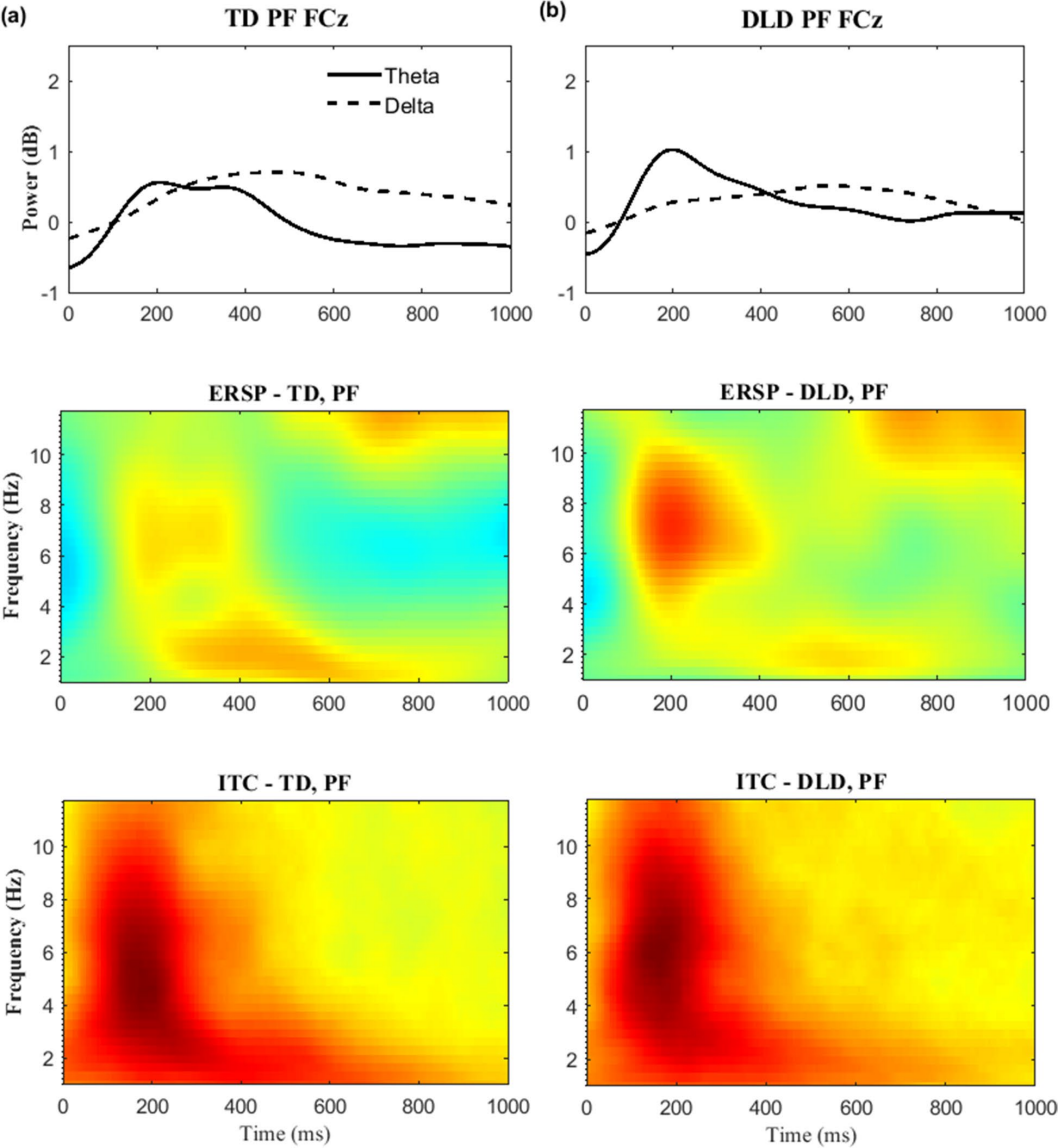
Time–frequency measures elicited by positive feedback (PF) at electrode FCz for children with TD (**a**) and DLD (**b**). On the top are filtered time–frequency waveforms; dotted lines represent the power of the delta frequency band (<3 Hz), and solid lines represent the theta frequency band (4–7 Hz). ERSP and ITC measures are presented in the middle and at the bottom, respectively

Much of the oscillatory activity during the FRN and P3a time window occurred in the *theta* band (4–7 Hz), as shown in Figs. 3 and 4, respectively. A significant feedback effect for *theta* was found in the FRN, *F* (1, 43) = 19.426, *p* = 0.001, *η*^*2* ^= 0.31, and P3a time windows *F* (1, 43) = 15.102, *p* = 0.001, *η*^*2*^ = 0.26, indicating stronger *theta* to negative than positive feedback in both groups. There was no evidence of a *group* effect [FRN: (*F* (1, 43) = 0.129, *p* = 0.722, *η*^*2* ^= 0.003; P3a: *F* (1, 43) = 0.055, *p* = 0.815, *η*^*2* ^= 0.001)] or a *feedback-by-group* interaction [FRN: (*F* (1, 43) = 0.028, *p* = 0.868, *η*^*2* ^= 0.001; P3a: *F* (1, 43) = 0.055, *p* = 0.815, *η *^*2* ^= 0.001)] in terms of *theta* power. For *delta* activity, no effect of feedback valence was found within the FRN or P3a time windows: *F* (1, 43) = 0.198, *p* = 0.658, *η*^*2* ^= *0.005*, and *F* (1, 43) = 0.020, *p* = 0.887, *η*^*2* ^= *0*, respectively.

Next, we examined ITC as a measure of the coherence of brain oscillations over time (trial-to-trial cortical synchronization). A significant main effect of feedback valence was identified for *theta* ITC, *F* (1, 43) = 5.055, *p* = 0.03, *η*^*2* ^= 0.105, demonstrating a tendency for greater trial-to-trial coherence for *theta* activity in response to negative feedback than positive feedback in both groups. There was no significant group effect on *theta* ITC (*F* (1, 43) = 0.116, *p* = 0.735, *η*^*2* ^= 0.003), nor was there an interaction between feedback and group (*F* (1, 43) = 0.732, *p* = 0.397, *η*^*2* ^= 0.017). *Delta* ITC revealed a significant main effect for feedback valence in the FRN time window, *F* (1, 43) = 5.531, *p* = 0.023, *η*^*2* ^= 0.114, indicating that the ITC of *delta* activity was higher in response to positive than negative feedback. ERSP results for *delta* did not mirror this finding. Similar to the *delta* ERSP findings, no group effect or feedback-by-group interaction was detected, *F* (1, 43) = 0.316, *p* = 0.577, *η*^*2* ^= 0.007, and *F* (1, 43) = 0.014, *p* = 0.914, *η*^*2* ^= 0*,* respectively.

### Associations between time–frequency- and time-domain measures

The results of the regression analyses that evaluated the contribution of *theta* and *delta* to FRN and P3a elicited by positive and negative feedback in each group (TD, DLD) are presented in Table 3 and are described below.

#### Children with TD

As shown in Table 3, no significant contribution of *theta* to FRN positive was found in the TD group, *β* =  − 0.281, *t* =  − 1.758, *p* > 0.05, and the contribution of *theta* to FRN negative approached significance, *β* =  − 0.407 *p* = 0.052. A strong contribution of *delta* was found to FRN positive in the TD group, *β* = 0.879, *t* = 5.503, *p* < 0.001, while the unique contribution from *delta* to FRN negative approached significance, *β* = 0.405, *p* = 0.054. While *delta* activity was found to contribute to the P3a positive, *theta* activity was not (*delta β* = 0.578, *p* < 0.05; *theta β* =  − 0.116, *p* > 0.05).

ITC measures indicated a contribution of consistent trial-to-trial phase locking of *theta* and *delta* activity to the FRN positive in the TD group (*delta* ITC *β* = 0.656, *p* < 0.001; *theta* ITC *β* =  − 0.478, *p* = 0.001) (see Table 4). Additionally, *delta* ITC was found to be coherent with the FRN negative while *theta* ITC did not (*delta β* = *0.592*, *p* < 0.01; *theta* β =  − 0.366, *p* > 0.05). Trial-by-trial phase locking of *theta* and *delta* activity to the P3a positive was not observed (*delta* ITC *β* = 0.053, *p* > 0.05; *theta* ITC *β* =  − 0.089, *p* > 0.05). While the model for the P3a to negative feedback was majorly predicted by *delta* activity, (*delta β* = *0.523*, *p* < 0.01; *theta β* = 0.303, *p* > 0.05) (see Table 3), the trial-by-trial phase locking of *theta* activity to the P3a negative was found to contribute significantly (*delta β* = *0.145*, *p* > 0.05; *theta β* = 0.548, *p* < 0.01) (see Table 4).

**Table 4:** Results of regression models using inter-trial coherence (ITC) measures of *delta* and *theta* to predict the FRN and P3a for group and feedback valence

**ITC regression models**													
		**TD**						**DLD**					
		**Negative**			**Positive**			**Negative**			**Positive**		
**Model**		**Beta**	**t**	**Semi P. Corr**	**Beta**	**t**	**Semi P. Corr**	**Beta**	**t**	**Semi P. Corr**	**Beta**	**t**	**Semi P. Corr**
**FRN**	**Delta**	0.592	3.118^**^	0.554	0.656	5.152^***^	0.739	0.198	0.542	0.13	0.355	1.427	0.327
	**Theta**	-0.366	-1.928	-0.38	-0.478	-3.756^***^	-0.625	-0.231	-0.633	-0.152	0.021	0.084	0.02
	**F**	5.193^*^			19.841^***^			0.205			1.302		
	**Adj. R2**	0.321			0.643			0.024			0.133		
**P3a**	**Delta**	0.145	0.782	0.164	0.053	0.249	0.053	-0.206	-0.97	-0.229	-0.255	-0.377	-0.215
	**Theta**	0.548	2.965^**^	0.534	-0.089	-0.418	-0.089	0.735	3.458^**^	0.643	0.348	1.237	0.287
	**F**	7.066^**^			0.111			6.397^**^			0.796		
	**Adj. R2**	0.391			0.01			0.429			0.086		

^*^Correlation is significant at the 0.05 level (2-tailed)

^**^Correlation is significant at the 0.01 level (2-tailed)

^***^Correlation is significant at the 0.001 level (2-tailed)

#### Children with DLD

There was no evidence that *delta* or *theta* activity significantly contributed to the FRN positive or negative in this group (*p* > 0.05) (see Table 3). The models predicting the P3a to positive feedback indicated a significant contribution from *theta* activity (P3a positive; *β* = 0.633, *p* < 0.01). The contribution from theta to P3a negative approached significance (P3a negative; *β* = 0.471, *p* = 0.06). *Delta* was not found to contribute to the P3a positive or negative in the DLD group.

ITC measures indicated no trial-to-trial phase locking of theta and *delta* activity to the FRN in the DLD group. However, a trial-by-trial phase locking of theta to the P3a negative was observed (theta ITC *β* = 0.735, *p* < 0.01) (see Table 4).

These results indicate that *theta* activity played a stronger role in the processing of feedback in the DLD group, whereas in the TD group, *delta* activity modulated the FRN and the P3a.

### Associations between EEG measures and behavioral indicators of performance

To evaluate the unique contribution of the *theta* and *delta* activities to the behavioral measures of performance, regression analyses were conducted (see Table 5). In children with TD, delta activity associated with positive and negative feedback in the FRN (FRN negative *β* = 0.545, *p* < 0.01; FRN positive *β* = 0.649 *p* < 0.01), and P3a (P3a negative *β* = 0.579, *p* < 0.01; P3a positive: *β* = 0.578, *p* < 0.05) time windows predicted test performance. However, no such associations between performance and *delta/theta* activity were found in children with DLD (see Table 5). These observations were consistent with and complemented by results from the ITC measure.

**Table 5:** Results of regression models using ERSP measures of *delta* and *theta* to predict performance accuracy for group and feedback valence

**Accuracy regression models**													
		**TD**						**DLD**					
		**Negative**			**Positive**			**Negative**			**Positive**		
**Model**		**Beta**	**t**	**Semi P. Corr**	**Beta**	**t**	**Semi P. Corr**	**Beta**	**t**	**Semi P. Corr**	**Beta**	**t**	**Semi P. Corr**
**FRN**	**Delta**	0.545	3.012^**^	0.54	0.649	3.197^**^	0.563	0.035	0.14	0.031	-0.299	-1.183	-0.256
	**Theta**	0.125	0.689	0.145	-0.144	-0.711	-0.15	-0.016	-0.064	-0.014	0.179	0.707	0.156
	**F**	6.086^**^			5.756^**^			0.01			0.706		
	**Adj. R2**	0.356			0.344			0.001			0.066		
**P3a**	**Delta**	0.579	3.427^**^	0.59	0.501	2.382^*^	0.453	0.047	0.183	0.041	-0.37	-1.418	-0.302
	**Theta**	0.173	1.021	0.213	0.078	0.369	0.078	0.05	0.193	0.043	0.359	1.375	0.294
	**F**	7.965^**^			4.682^*^			0.07			1.228		
	**Adj. R2**	0.42			0.299			0.007			0.109		

^*^Correlation is significant at the 0.05 level (2-tailed)

^**^Correlation is significant at the 0.01 level (2-tailed)

^***^Correlation is significant at the 0.001 level (2-tailed)

### Summary of the results

Our results indicate that learning outcomes were poorer among children with DLD. Electrophysiological measures of feedback processing in the time domain indicated that negative feedback provided to children with DLD elicited no FRN and P3a amplitude differences between positive and negative feedback when compared with the TD group. In the time–frequency domain, the contribution of the feedback-related *delta* and *theta* to the FRN, P3a, and to learning outcomes were found to differ between groups. *Theta* and *delta* activities uniquely contributed to the FRN and P3a, with an overall stronger contribution from *delta* in the TD group and *theta* in the DLD group. Interestingly, *delta* activity to both negative and positive feedback in the FRN and P3a time windows was found to correlate with performance accuracy, but only in the TD group.

## Discussion

The results reveal differences between children with DLD and their TD peers in learning outcomes and in the electrophysiological measures of feedback processing at the time and time–frequency domains. In line with previous findings, negative feedback provided to children with typical development elicited larger FRN and P3a amplitudes than positive feedback (e.g., [2–4, 30, 32, 43]. Expected differences between the amplitudes of the FRN and P3a in response to positive and negative feedback were only found in the TD group and were absent in the DLD group. These results are in line with previous reports [1, 2] and may indicate an ineffective distinction between the information provided by positive and negative feedback among children with DLD.

Evidence from previous studies suggests that the FRN reflects a composite signal comprised of separable negative feedback-related theta and positive-feedback related delta activities [12, 35, 89] indexing distinct processes stemming from separate neuronal sources [48], such that negative feedback-related theta activity is reported to stem from the ACC [20, 35, 45] and a positive feedback-related delta activity is suggested to emerge from the striatum [10, 12, 13, 18]. It is proposed that while post-feedback mid-frontal *theta* activity may be crucial for detecting errors and negative outcomes [19, 61], subsequence performance adjustment is driven by *delta* activity. When evaluating the frequency makeup of the FRN and P3a in the TD and DLD groups in the present study, a pattern emerged that may reflect processing differences between the two groups. More specifically, in the TD group, the electrophysiological measures of feedback processing in the time-domain (i.e., FRN and P3a) were dominated by *delta* activity, which was also linked to behavioral indicators of performance. In children with DLD, on the other hand, it was the *theta* activity that was found to dominate the FRN, but such activation was not a predictor of learning outcomes. Children with DLD did not show the same delta activity that was observed in children with TD or the relationship between delta activity and performance. A similar pattern of results was found for the phase coherence (ITC) measure, revealing that inter-trial delta synchrony not only predicted the variance in the FRN and P3a but also strongly correlated with the behavioral outcomes in the TD group. No such pattern of results was observed in children with DLD.

The current results provide evidence to suggest that the FRN and P3a reflect a blend of *theta* and *delta* activities. Interestingly, *delta* activity, which is typically associated with reward processing, was found to contribute to both negative and positive feedback processing and to be a strong predictor of learning outcomes in the TD group. Similar results were found by Bowers et al. [17], who reported that only *delta* power contributed to the FRN in adolescents while they were engaged in a gambling task [17, 36]. In a separate study using the same task with older participants, both delta and theta power were observed to account for the variance in the FRN [35]. It is possible that the dominance of *delta* activity in the TD group in the present study can be explained within the context of developmental changes, given evidence of age-related differences in feedback-related oscillatory activity [17] and maturation differences between the striatum which is assumed to be the source of the feedback related *delta* activity and the ACC, which is the source of the feedback-related *theta* activity [35, 42, 45, 46, 62]. It is possible that *theta*-driven processing of negative feedback is ineffective in children because of the immaturity of the ACC. Evidence in support of this suggestion comes from the evaluation of behavioral adjustments following feedback in children. More specifically, in previous reports [2, 43, 59], children’s stay behavior (i.e., repeating a correct response following positive feedback) was found to be above chance level, whereas their switch behavior (changing a behavior following negative feedback) was found to be at or below chance level. These results may be interpreted to reflect ineffective processing of negative feedback in children that may be attributed, at least in part, to an immature executive system in the ACC.

Another possible explanation for the dominance of *delta* in the TD group in the current study is the probabilistic nature of the learning paradigm that may rely on striatum activation. The striatum, which has been reported to activate in anticipation of reward in both children and adults [39] and is proposed to be the generator of feedback-related delta activity [10, 18, 36] has been found to play a crucial role in non-declarative feedback-based learning [9, 69, 70, 93]. It is possible that the input of *delta* and *theta* to the feedback-related ERPs reflects the extent to which their sources contribute to the processing as a function of the nature of the learning paradigm.

Our data suggest that it is the *delta* activity that predicts learning outcomes in children with TD and may account for individual differences in feedback-based learning in children. This assertion is based on previous suggestions that feedback-related mid frontal *theta* activity, as part of ACC’s monitoring system, indexes an initial assessment of the primary attributes of feedback, whereas *delta* power is more sensitive to secondary or more complex aspects of feedback processing [12, 89]. The lack of *delta* contribution to the feedback-related ERPs or learning outcomes in the DLD group can be interpreted as evidence of striatal dysfunction in this group. This suggestion is in line with a previous report of an association between low *delta* power and impaired reward sensitivity and basal ganglia dysfunction in individuals with psychological distress [35] and can be supported by evidence of impaired implicit learning [40, 53, 96] as well as striatal abnormalities in children with DLD [7, 50, 58, 60, 77, 79, 87, 88]. The results of the study can be interpreted to suggest that post-feedback atypical *delta* activity is linked to impaired striatum-based processing of feedback in children with DLD.

## Conclusion

*Theta* and *delta* activities uniquely contributed to the FRN and P3a, with an overall stronger contribution from *delta* in the TD group and *theta* in the DLD group. While negative feedback elicited stronger *theta* activity in comparison to positive feedback in both groups, only *delta* activity was found to be associated with performance accuracy, but only in the TD group. The results suggest that processing and possibly performance differences between the two groups can be attributed to the lack of striatum-based delta contribution to feedback processing in children with DLD.

## Data Availability

The datasets during and/or analyzed during the current study are available from the corresponding author upon reasonable request.
